# Telomerase reverse transcriptase promotes chemoresistance by suppressing cisplatin-dependent apoptosis in osteosarcoma cells

**DOI:** 10.1038/s41598-017-07204-w

**Published:** 2017-08-01

**Authors:** Zhengpei Zhang, Ling Yu, Guo Dai, Kezhou Xia, Gaiwei Liu, Qi Song, Chunjie Tao, Tian Gao, Weichun Guo

**Affiliations:** 1 Department of Orthopedics, Renmin Hospital of Wuhan University, Wuhan, Hubei China; 2 Key Laboratory of Carcinogenesis and Translational Research, Ministry of Education, Department of Orthopedic Oncology, Peking University Cancer Hospital& Institute, Beijing, China

## Abstract

Cisplatin is one of the most efficacious antimitotic drugs used in the treatment of a range of malignant tumors. However, treatment failures are common due to the development of chemoresistance. In addition to its telomere maintenance function, telomerase plays a pro-survival role, inducing decreased apoptosis and increased resistance against DNA damage. Elucidation of the molecular mechanisms underlying this effect is critical to improve treatment outcomes. Previously, our group showed higher telomerase reverse transcriptase(TERT) expression in cisplatin resistant osteosarcoma cells. In this study, confocal fluorescence microscopy experiments revealed that TERT translocates from the nucleus to mitochondria in cisplatin treated osteosarcoma cells. We observed decreased apoptosis rate and improved mitochondrial function in TERT-overexpressing cells following cisplatin treatment. Based on these results, we further established that TERT inhibits cisplatin-induced apoptosis independently of telomerase reverse transcriptase activity. Moreover, TERT suppressed cisplatin-induced apoptosis and improved mitochondrial function via alleviating intracellular ROS in osteosarcoma cells. Our finding that TERT shuttles from the nucleus to the mitochondrion in response to cisplatin treatment and inhibits cisplatin-induced apoptosis in osteosarcoma cells may be especially important to overcome drug resistance.

## Introduction

Osteosarcoma, the most commonly diagnosed primary malignant bone tumor type, is highly aggressive, with a peak incidence in adolescents^1, 2^. The introduction of adjuvant and neoadjuvant chemotherapy has greatly prolonged survival, and limb salvage surgery has significantly improved the quality of life of osteosarcoma patients^3^. However, many patients are insensitive to the currently available chemotherapeutic agents and have poor prognosis^4^. Elucidating of the mechanisms underlying chemo-resistance is therefore essential to improve outcomes.

Telomerase is composed of protein and RNA components, the major being telomerase reverse transcriptase (TERT) and its RNA partner TERC^5^. Telomerase expression is associated with cell immortalization and tumorigenesis^6^. Elongation of telomeres is considered the prime function of reactivated TERT. However, this activity does not account for all its effects; such as cell growth, proliferation and resistance to apoptosis^7–9^. For instance, expression of TERT in some human and murine cell types causes rapid cell proliferation, with no accompanying measurable changes in telomere elongation^10–12^. Inhibition of TERT via an antisense strategy can trigger short-term apoptosis, without affecting telomere length^13, 14^. These results indicate that TERT affects cell survival via pathways independent of telomere erosion^15^.

Apoptosis results from a sequence of events involving a number of gene products, including the survival factors Bcl-2, Bcl-xl and Bax, which are members of the Bcl-2 family. Expression of Bcl-2 and Bcl-xl has been shown to suppress apoptosis, whereas Bax promotes apoptosis in response to different stimuli^16–18^. These survival factors have been shown to play important roles in the mitochondrial apoptosis pathway^19, 20^. Furthermore, Bcl-2 family members have been implicated in the regulation of telomerase activity, suggesting a close link between Bcl-2 proteins and telomerase^21, 22^.

Our data clearly demonstrate that TERT significantly inhibits cisplatin-induced apoptosis in osteosarcoma cells in correlation with its mitochondrial translocation. Consistent with our findings, Buchner *et al*. showed that TERT in the nucleus is exported to mitochondrial under conditions of oxidative stress in a Src kinase dependent manner^23^. Moreover, we prove that this protective role of TERT is independent of telomerase reverse transcriptase activity. Specific mechanism is clarified that TERT reduces cisplatin-induced apoptosis by alleviating intracellular ROS levels. This study provides a novel insight into the mechanism whereby TERT suppresses cisplatin-induced apoptosis and may open a new way to solving osteosarcoma chemo-resistance.

## Results

### TERT translocates from the nucleus to mitochondria following cisplatin treatment in osteosarcoma cells

**Figure 1:**
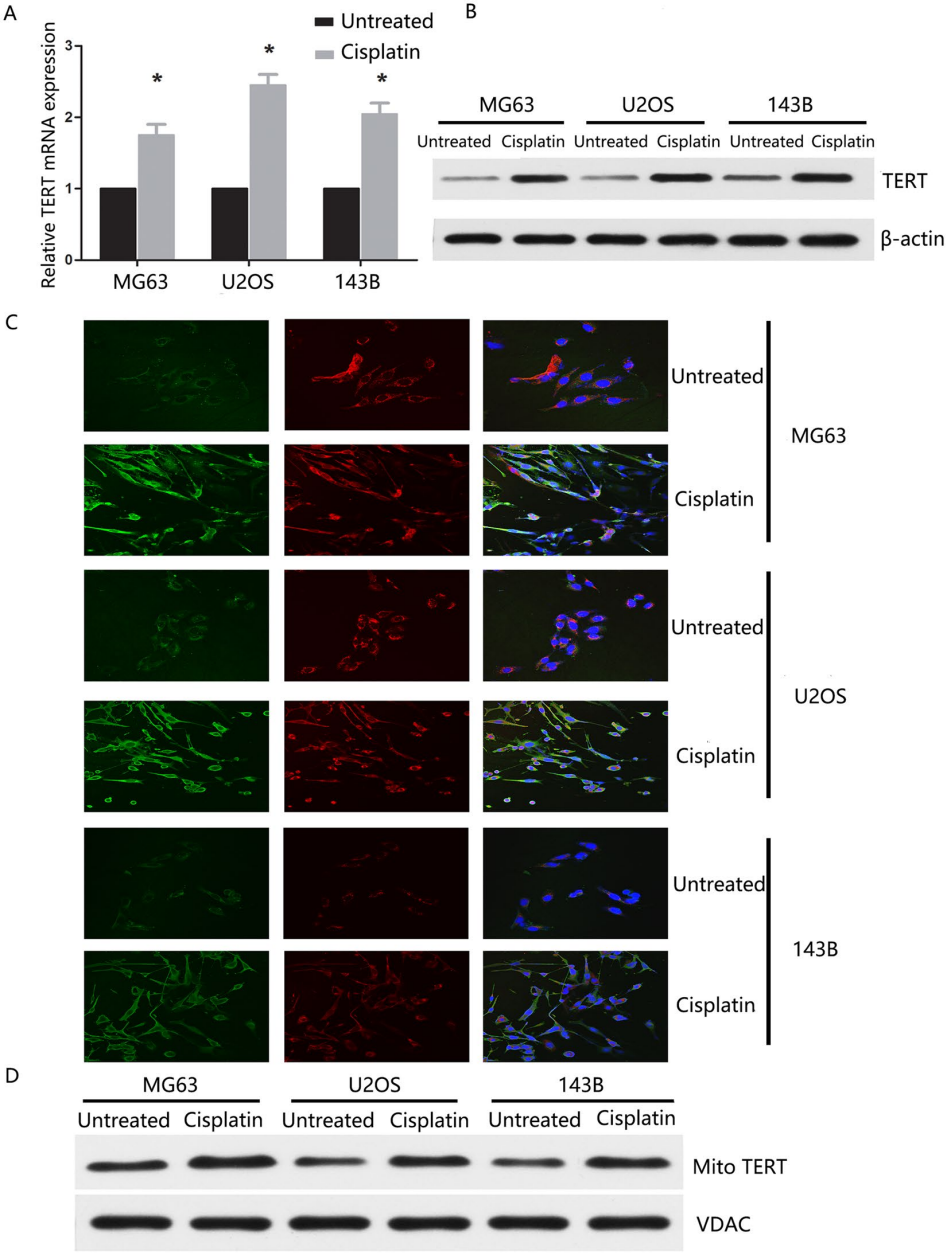
TERT expression is altered and shuttles from the nucleus to mitochondria in cisplatin-treated osteosarcoma cells. (**A**) The relative mRNA expression of TERT were determined by qRT-PCR in cisplatin treated or untreated osteosarcoma cells including MG63, U2OS and 143B. (**B**) The relative protein expression of TERT were analyzed by Western-blot in cisplatin treated or untreated osteosarcoma cell lines. (**C**) Confocal images from MG63, U2OS and 143B cells untreated (control, upper panel) or treated with 5 μmol/L cisplatin for 24 h (lower panel). Green anti-TERT immune-fluorescence, red mitotracker and blue nuclear DNA (DAPI). Marked colocalization between green anti-TERT and red mitotracker is displayed by green-red mixing. (**D**) Mitochondrial fractions of cisplatin treated or untreated cells were subjected to Western blot analysis of TERT and VDAC. All experiments were carried out at least triplicates and the data were presented as the mean ± S.D. Student t test was performed to evaluate the difference *P < 0.05.

To determine whether TERT expression is altered after treatment with cisplatin, we initially analyzed TERT mRNA levels in osteosarcoma cell lines MG63, U2OS and 143B. TERT mRNA expression was significantly higher in cisplatin-treated (5 μmol/L, 24 h) osteosarcoma cells, compared to untreated cells (Fig. 1A). Western-blot revealed significantly elevated TERT protein in osteosarcoma cells treated with cisplatin relative to untreated cells, consistent with mRNA data (Fig. 1B). Subcellular shuttling of TERT protein from the nucleus to mitochondria has been reported in different cell types, including human cervical cancer, human neuroblastoma and human breast adenocarcinoma cells^24–27^. Accordingly, we investigated whether this shuttling of endogenous telomerase occurs in osteosarcoma cells after cisplatin treatment. MG63, U2OS and 143B cells treated with cisplatin exhibited robust TERT expression in mitochondria, as determined with confocal fluorescence microscopy (Fig. 1C). Interestingly, we observed minimal alternations of TERT in MG63, U2OS and 143B cells treated with 2.5 μmol/L cisplatin for 24 hr. However, TERT was strongly expressed at both mRNA and protein levels in all three osteosarcoma cells treated with 5 μmol/L cisplatin for 24 h in keeping with our previous *in vitro* finding of TERT enrichment in chemo-resistant osteosarcoma cells^28^. This mitochondrial localization pattern of TERT was further detected by Western-blotting and similar results were obtained (Fig. 1D).

### TERT inhibits cisplatin-induced apoptosis in osteosarcoma cells

**Figure 2:**
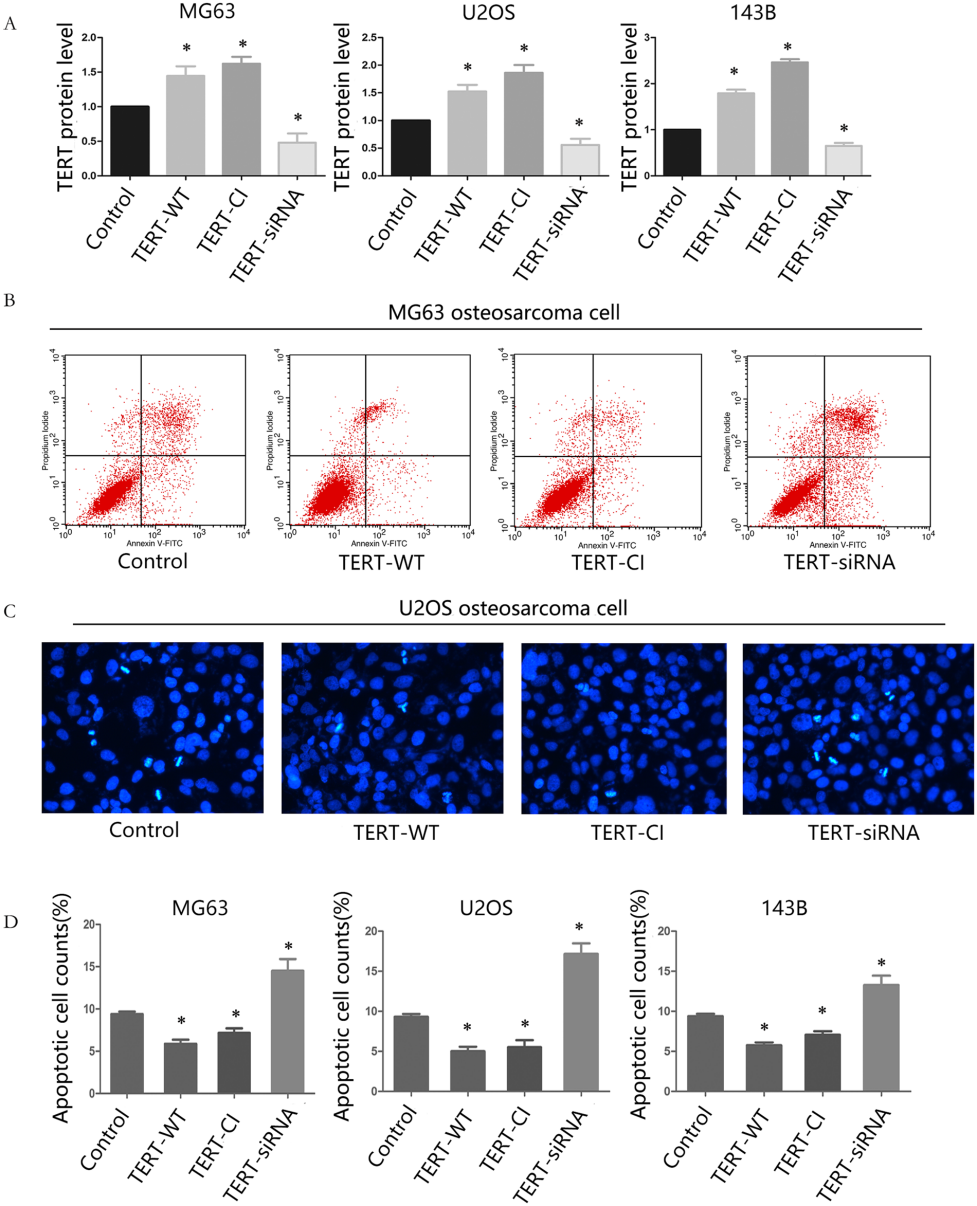
TERT reduces apoptosis induced by cisplatin in osteosarcoma cells independently of telomerase reverse transcriptase activity. (**A**) The relative TERT expression in mock transfected cells (control), TERT-overexpressing cells (TERT-WT, TERT-CI) and TERT-siRNA cells were assessed by Western blot analysis. (**B**) MG63 transfected cells were treated with 5 μmol/L cisplatin for 24 hours after which the apoptosis in cells was detected by flow cytometry. (**C**) U2OS transfected cells were treated wit with 5μmol/L cisplatin for 24 hours after which the apoptosis in cells was assessed by Immuno-fluorescence. Karyopyknosis is taken as apoptosis. (**D**) Quantification of apoptotic rate in cisplatin-treated osteosarcoma transfected cell lines detected by FACS. All experiments were carried out at least triplicates and the data were presented as the mean ± S.D. Student t test was performed to evaluate the difference *P < 0.05.

To investigate the impact of TERT expression on cisplatin-induced apoptosis, osteosarcoma cells were stably transfected and separated into four groups, specifically, TERT-wildtype (TERT overexpressing cells), catalytically-inactive TERT (TERT overexpression cells with inactive telomerase reverse transcriptase), TERT-siRNA and negative control (mock-transfected cells). As to test and verify the transfection effect, we detected TERT expression and found it increasingly expressed in the TERT-wildtype (TERT-WT) and catalytically-inactive TERT (TERT-CI) cells and decreasingly expressed in siRNA-transfected cells (Fig. 2A). As expected, TERT expression were observed as no differences between TERT-WT and TERT-CI cells. Stably transfected osteosarcoma cells were treated with cisplatin for 24 h and apoptosis rate were analyzed by flow cytometric. Double staining with annexin V-fluorescein isothiocyanate(FITC) and propidium iodide(PI) revealed a marked decrease in TERT-WT and TERT-CI cells. While apoptosis of TERT-siRNA transfected cells was significantly increased, compared to that in control MG63 osteosarcoma cells (Fig. 2B). Similar results were obtained with Immuno-fluorescence analysis (Fig. 2C). Flow cytometry was applied to quantify the percentage of apoptotic cells from all three transfected osteosarcoma cell lines after cisplatin treatment. An approximate two-fold decrease in apoptosis was calculated in TERT-WT and TERT-CI cells and a 1.5-fold increase in TERT-siRNA transfected cells. (Fig. 2D). Moreover, no marked differences were evident between TERT-WT and TERT-CI transfected cells, suggesting that this anti-apoptotic effect does not rely on telomerase reverse transcriptase activity.

### TERT protects osteosarcoma cells against cisplatin-induced apoptosis through the mitochondrial pathway

**Figure 3:**
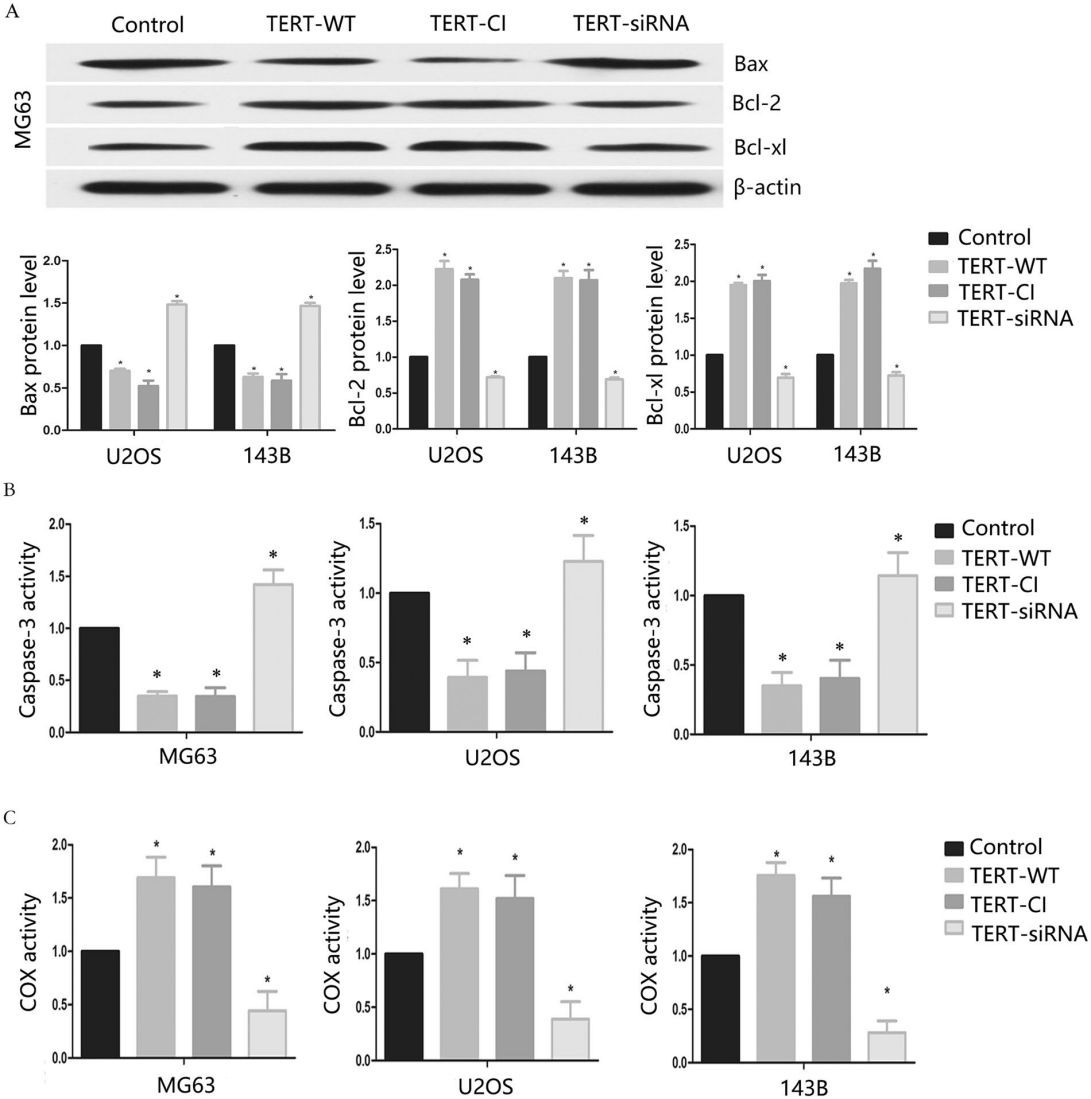
TERT inhibits mitochondrial apoptotic pathway in cisplatin-treated osteosarcoma cells. (**A**) proapoptotic Bax expression and antiapoptotic Bcl-2, Bcl-xl expression in transfected osteosarcoma cells were assessed by Western blot analysis. (**B**) Caspase-3 activity in transfected osteosarcoma cells was assayed using a caspase assay kit, following the manufacture’s protocol. (**C**) Transfected osteosarcoma cells were subjected to COX activity assay, spectrophotometrically determining the rate of COX activity through the addition of reduced cytochrome c. All experiments were carried out at least triplicates and the data were presented as the mean ± S.D. Student t test was performed to evaluate the difference *P < 0.05.

In view of TERT translocation to mitochondria (demonstrated using confocal fluorescence microscopy), we hypothesized that TERT inhibits cisplatin-induced apoptosis through the mitochondrial pathway. To confirm this theory, we detected expression of antiapoptotic Bcl-2, Bcl-xl and proapoptotic Bax proteins, which regulated the mitochondrial pathway. In all three cell lines, Bcl-2 and Bcl-xl protein levels were significantly increased and Bax protein was decreased in TERT-WT and TERT-CI transfected groups, while opposite expression patterns were observed in TERT-siRNA transfected cells (Fig. 3A). Caspase family members have been reported as an important downstream regulators in the mitochondrial pathway of apoptosis^29^. In our study, TERT significantly inhibited caspase-3 activity in osteosarcoma cells (Fig. 3B), but exerted no obvious effects on caspase-9 activity (data not shown). Given the localization of TERT into mitochondria coupled with the functional significance of COX in mitochondrial respiration, we focused on the role of TERT in mitochondrial respiration in relation to COX. Cells transfected with wildtype and catalytically inactive TERT maintained higher COX activity, and conversely, TERT-siRNA transfected cells showed lower COX activity, compared with control cells (Fig. 3C). The results collectively indicated that TERT significantly suppresses expression of mitochondrial apoptosis-related proteins.

### TERT inhibits apoptosis induced by cisplatin through alleviating intracellular ROS in osteosarcoma cells

**Figure 4:**
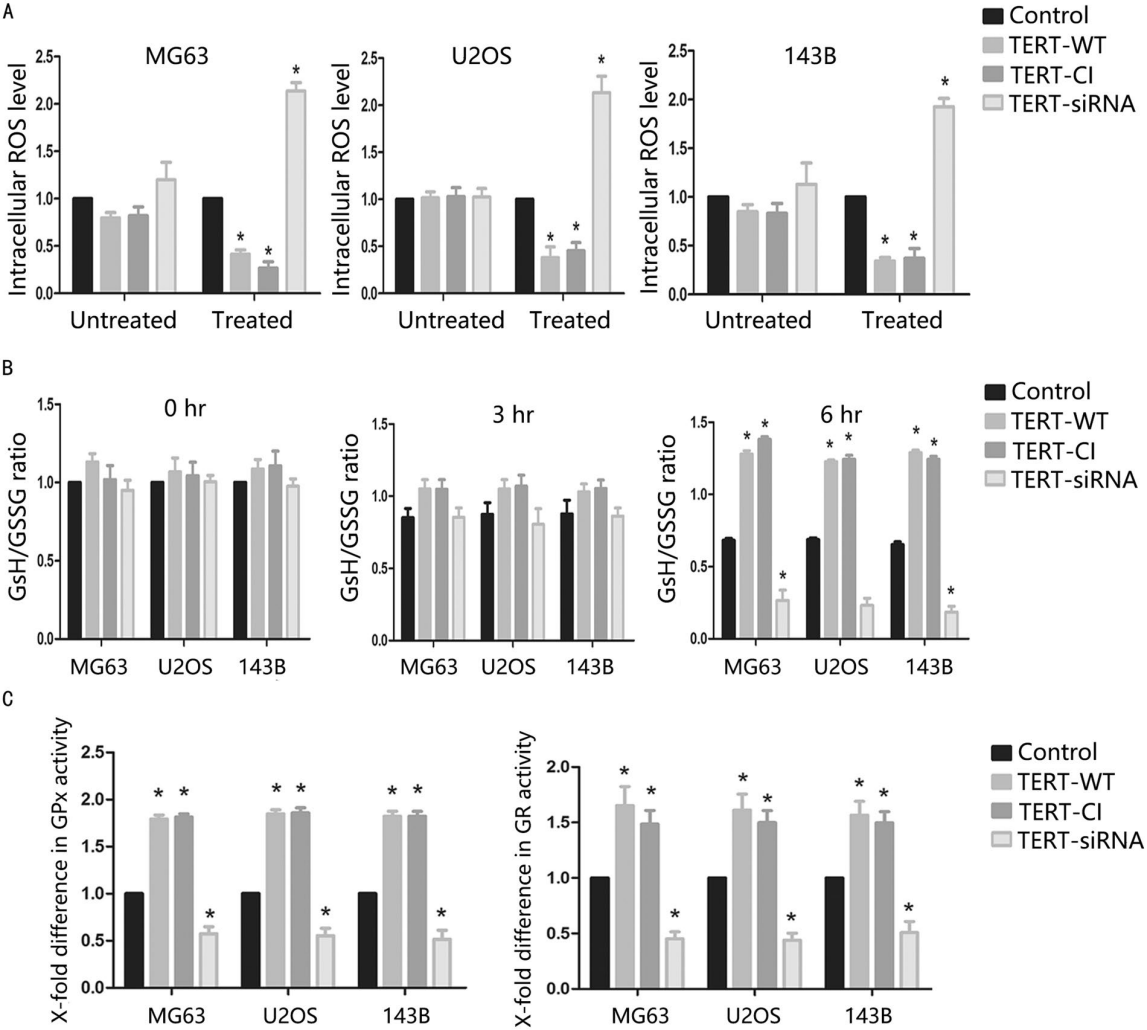
TERT enhances cisplatin-resistance in osteosarcoma cells via promoting glutathione antioxidant defense system to reduce ROS level. (**A**) Transfected osteosarcoma cells were exposed to 5 μmol/L of cisplatin for 24 hours following which cellular ROS was assessed using the probe DCHF-DA. (**B**) Transfected osteosarcoma cells were treated with 5 μmol/L of cisplatin from 0 to 6 hours following which intracellular GSH/GSSG ratio was computed. (**C**) Transfected osteosarcoma cells were used to prepare the sample for the GPx and GR activity assay, and the fold difference was calculated. Data shown are mean ± S.D. Student t test was performed to evaluate the difference *P < 0.05.

We hypothesized that translocated TERT promotes resistance of osteosarcoma cells to cisplatin-induced intracellular ROS production. To examine this possibility, we investigated the ROS response of TERT-WT, TERT-CI and siRNA transfected cells to cisplatin treatment. Flow cytometric analysis revealed significantly lower basal and mitochondrial ROS levels in TERT-WT and TERT-CI transfected cells, and conversely, higher ROS levels in TERT siRNA transfected cells. The G-mean values of dichlorofluorescein fluorescence obtained from three independent experiments were used to calculate the fold difference in ROS levels among the cell groups (Fig. 4A). To provide further insight into this apoptosis effect of TERT, we performed glutathione assay measuring GSH/GSSG couple. Glutathione antioxidant defense capacity was investigated to determine if TERT influenced this redox couple, thus altering the cellular capacity to resist oxidative stress. Specifically, TERT-WT, TERT-CI, TERT-siRNA and control cell groups were exposed to cisplatin from 0 to 6 h and assayed for their intracellular ratios of GSH /GSSG. A higher GSH/GSSG ratio was determined in TERT-WT and TERT-CI cells and a lower ratio in TERT-siRNA cells, compared with control cell group (Fig. 4B). Next, the activities of glutathione peroxidase(GPx) and glutathione reductase(GR), important regulators of glutathione levels, were assessed. Higher GPx and GR activities were maintained in TERT-WT and TERT-CI cells, while TERT-siRNA cells showed lower activity, compared with control cells (Fig. 4C). Our results suggested that TERT reduces intracellular ROS levels, and further alleviated apoptosis by inhibiting mitochondrial apoptotic pathway in cisplatin-treated osteosarcoma cells.

### TERT enhances cisplatin resistance of osteosarcoma cells *in vivo*

**Figure 5:**
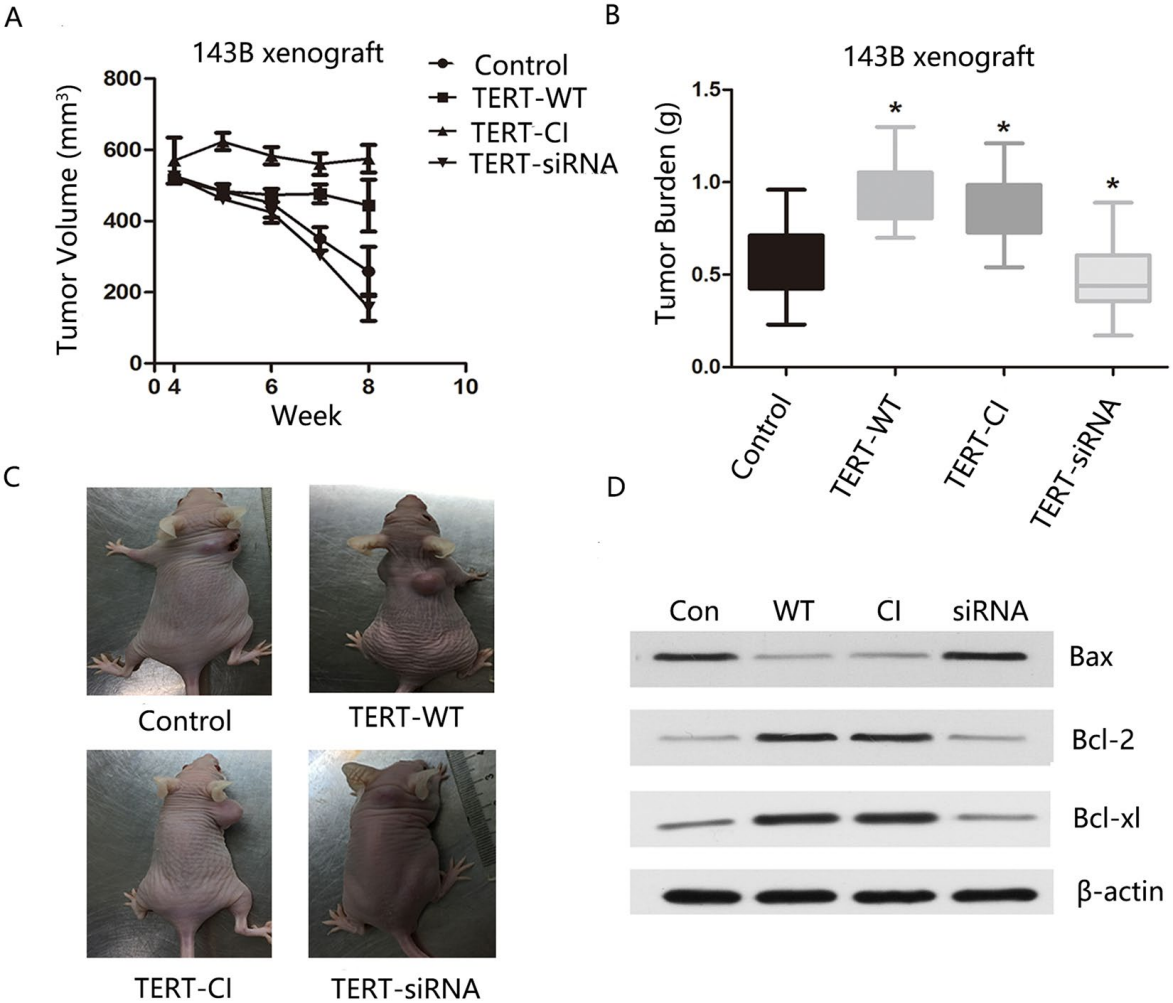
Cisplatin-resistance of osteosarcoma cells is promoted by TERT in vivo. (**A**) BALB/C nude mice with transfected xenograft tumors of 500 mm^3^ were given 5 mg/kg of cisplatin at embedded intervals of 4 days for 4 weeks. TERT-overexpressing xenograft tumors showed more resistant to cisplatin treatment. Data are represented as mean ± SEM *p < 0.05. (**B**,**C**) Tumor burden were assessed at time of death (8 weeks). Results expressed as mean ± 95% confidence intervals. n = 6 *p < 0.05. (**D**) Proteins were extracted from tumor burden. Apoptotic-related proteins expression were then assessed by Western blot analysis. β-actin was used as a leading control.

**Figure 6:**
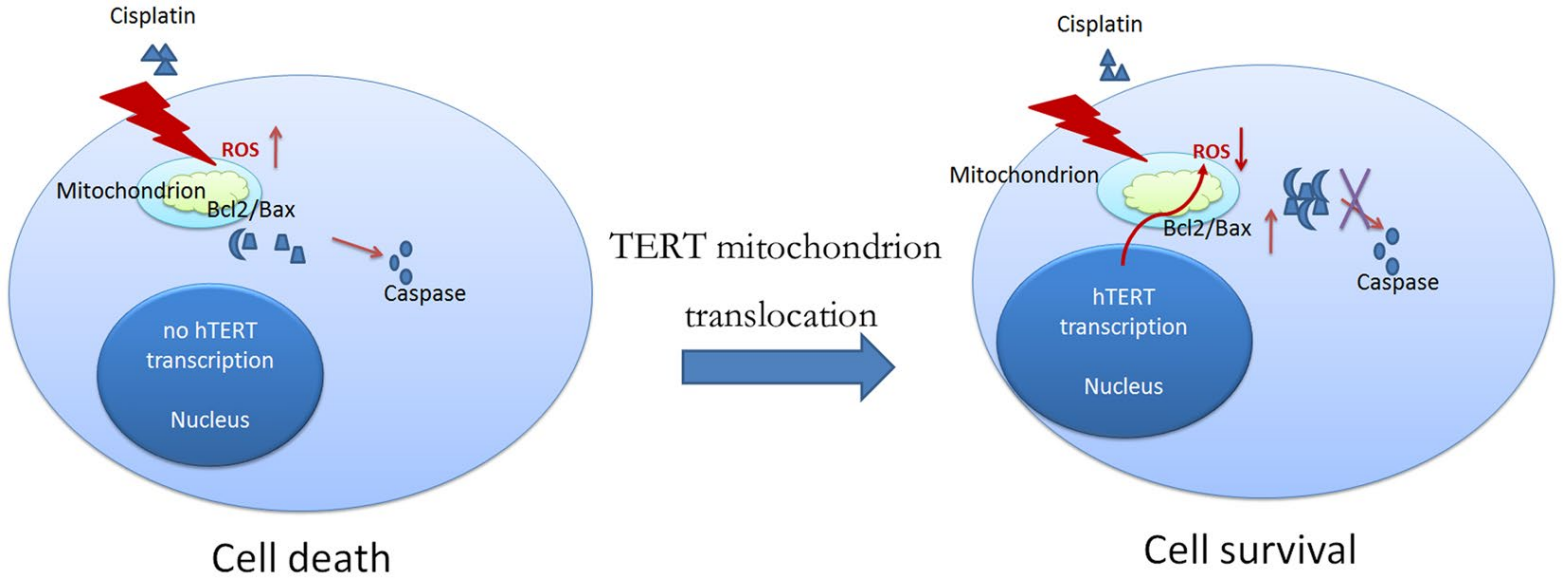
Underlying mechanism of TERT-mediated protection against cisplatin-induced osteosarcoma cell death. In cisplatin sensitive osteosarcoma cells, TERT doesn’t shuttles from the nucleus to mitochondrion upon cisplatin treatment, which results elevated intracellular ROS levels. Increased ROS levels further activates mitochondrial apoptotic pathway and causes programmed cell death. In cisplatin-resistant osteosarcoma cells, TERT shuttles from the nucleus to mitochondrion and alleviates intracellular ROS levels upon cisplatin treatment, which results inhibited mitochondrial apoptosis and cell survival.

To establish whether TERT promotes cisplatin resistance *in vivo*, we subcutaneously inoculated tumor xenografts derived from 143B cells into NOD/SCID mice. Mice bearing tumors of 10 mm in diameter (500 mm^3^ in volume) (n = 6) were injected intraperitoneally with 5 mg/kg cisplatin at intervals of 4 days for 4 weeks. Tumors of mice inoculated with control cells grew slowly and reached a mere 600 mm^3^ after 4 weeks. TERT-WT and TERT-CI inoculated tumors displayed no obvious differences relative to the initially implanted size. TERT-siRNA treated tumor cells showed the slowest growth, displaying the smallest sizes after 4 weeks. (Fig. 5A). All mice were euthanized and tumor burdens assessed at the time of death (8 weeks). TERT-WT and TERT-CI treated tumors displayed the largest volumes, while TERT-siRNA treated tumors showed smallest volumes than the control tumor group (Fig. 5B,C). Intrigued by these *in vivo* findings, we investigated whether the corresponding apoptotic proteins showed analogous changes in expression to data obtained *in vitro*. Western-blot analysis revealed that the Bax protein level stayed the lowest in TERT-WT and TERT-CI treated tumors and highest in TERT-siRNA treated tumors, compared with control tumors. Conversely, Bcl-2 and Bcl-xl protein levels remained the highest in TERT-WT and TERT-CI tumors and lowest in TERT-siRNA inoculated tumors, compared with control tumors (Fig. 5D). Based on the above results, we generated a schematic diagram to explain the mechanism underlying TERT-mediated protection against cisplatin-induced cell death (Fig. 6).

## Discussion

Cisplatin is one of the leading drugs used for conventional osteosarcoma chemotherapy. However, resistance to cisplatin is common in clinical therapy and the underlying mechanisms remain to be established. The recent discovery of the novel anti-apoptotic role of TERT, independent of its telomerase activity has provided a new perspective on antitelomerase and anti-TERT strategies^30^. Data from the current study provides strong evidence that constitutive TERT expression leads to not only reduced intracellular ROS levels, but also significant inhibition of the mitochondrial pathway of apoptosis induced by cisplatin, promoting resistance of osteosarcoma cells to cisplatin therapy.

To comprehensively understand the chemoresistance effects of TERT, we initially investigated TERT expression patterns in osteosarcoma cells subjected to cisplatin treatment. qRT-PCR and Western-blot demonstrated that cisplatin treated osteosarcoma cells display significantly higher TERT expression compared with control cells, consistent with results from our previous study^28^. Mitochondrial translocation of TERT is reported to endow cancer cells with the ability to evade death stimuli^31, 32^. Accordingly, we hypothesized that increased expression of TERT in cisplatin treated osteosarcoma cells promotes its translocation from the nucleus to cytoplasm. Confocal fluorescence microscopy disclosed translocation of TERT from the nucleus to mitochondria in all three osteosarcoma cell lines examined, clearly confirming our theory. Similar results were obtained by Chatchawan^33^, who demonstrated that mitochondrial telomerase protects cancer cells from nuclear DNA damage and apoptosis.

Mitochondria are the scene of a critical ‘point of no return’ in apoptosis^34^. When a cell receives a death signal, a’killer’ protein perforates in the membrane of the mitochondria. The membrane pores allow toxic molecules to leak out from mitochondria into the interior of the cell, where they trigger a series of events leading to dismantling of the cell from the inside. A number of mitochondrial proteins and pathways are involved in mitochondrial dependent apoptosis mechanisms, including the proapoptotic protein, Bax, and antiapoptotic proteins, Bcl-2 and Bcl-xl^35, 36^. The balance of proapoptotic and antiapoptotic members controls the sensitivity of cells to apoptosis. To determine whether TERT plays a role in the mitochondrial pathway of apoptosis, we established stable TERT-overexpressing and -depleted osteosarcoma cell lines (Supplementary Fig. S1). Our results suggest that constitutive TERT expression antagonizes cisplatin-induced apoptosis via regulation Bcl-2, Bcl-xl, Bax and caspase-3 in osteosarcoma cells. We propose that telomerase reverse transcriptase activity is not required for TERT-mediated inhibition of cisplatin-induced apoptosis, in view of the finding that a catalytically inactive TERT was as efficient as wildtype TERT in antagonizing cisplatin-induced apoptosis. This finding is consistent with earlier studies showing that TERT inhibits p53-induced apoptosis independently of telomerase reverse transcriptase activity^37^, and that telomere-independent activities of TERT can contribute to tumor development^38^. Smith *et al*.^12^ reported that wild-type TERT, but not a catalytically inactive form, was able to enhance cell growth under equivalent conditions Therefore, it is possible that TERT has multiple functions in cell growth and survival ability, some requiring (and others independent of) telomerase reverse transcriptase activity.

The precise molecular mechanisms underlying the ability of TERT to antagonize cisplatin-induced apoptosis are yet to be determined. Constitutive expression of TERT in osteosarcoma cells resulted in decreased level of intracellular ROS. we further observed that oxidized glutathione (GSH:GSSG) defense capacity and mitochondrial respiration function are enhanced in TERT-overexpressing cells. *In vitro* data were confirmed by the finding that TERT antagonizes cisplatin-induced apoptosis in a 143B xenograft. Based on the collective data, we generated a schematic diagram of the potential mechanism by which TERT suppresses cisplatin-induced apoptosis (Fig. 6). We proposed that cisplatin treatment enhanced intracellular ROS levels and activates the mitochondrial apoptotic pathway in cisplatin-sensitive osteosarcoma cells, leading to cell death. In cisplatin-resistant osteosarcoma cells, TERT translocates to in mitochondria, alleviates cisplatin-induced ROS levels and inhibits the mitochondrial pathway of apoptosis, promoting cell survival. In view of the above results, we considered whether TERT expression has an impact on prognosis of osteosarcoma patients. Using publicly available dataset, GSE38135, we assessed the potential role of TERT in metastasis occurrence and survival rate. Higher TERT expression was clearly evident in patients with metastasis (Supplementary Fig. S1A). In terms of survival differences between OS patients with high and low TERT expression, percentage survival was significantly lower in OS patients from the high expression group (Supplementary Fig. S2B).

To our knowledge, this study is the first to demonstrate that TERT antagonizes cisplatin-induced apoptosis independently of telomerase reverse transcriptase activity. Our data partially explain chemo-resistance in patients receiving cisplatin-based treatment. Since TERT plays an important role in cisplatin-resistance in osteosarcoma, we believe that suppressing its expression may contribute to eliminating cisplatin-resistant cells which was considered stem cells in our previous study^28^. Our findings suggest that inhibition of TERT may provide an effective therapeutic approach to eliminate cisplatin-dependent apoptosis in osteosarcoma cells, supporting its utility as a target for further clinical development.

## Materials and Methods

All lab protocols were approved by the Central Laboratory, Renmin Hospital of Wuhan University. All animal experiments were performed in accordance with Wuhan University Center for Animal Experiment (approval number S01315022l).

### Cell Culture

The human osteosarcoma cell lines MG63, U2OS and 143B were obtained from China Centre for Type Culture Collection (CCTCC). U2OS and MG63 cells were cultured in DMEM supplemented 10% FBS and 1% antibiotics (penicillin/streptomycin). 143B cells were cultured in MEM supplemented 10% fetal bovine serum (FBS) and 1% antibiotics (penicillin/streptomycin). Cells were propagated in a humidified environment at 37 °C with 5% CO_2_ and 100% humidity. Cell viability was determined using Trypan blue staining. Culture medium was replaced every two days.

### Generation of Stably Transfected Cell Lines

pBABE-puro-DN-hTERT was a gift from Bob Weinberg (Addgene plasmid # 1775)^39^, pBABE-puro-hTERT was a gift from Bob Weinberg (Addgene plasmid # 1771)^40^, and the indicated segments were cloned into lentiviral expression vector GV308 (Shanghai GeneChem). pMKO.1 puro TERT shRNA was a gift from William Hahn (Addgene plasmid # 10688)^7^, and the indicated segments was cloned into lentiviral RNAi vector GV329 (Shanghai GeneChem). Lentiviruses were created with the lentiviral vectors using standard techniques. To generate stably transfected cell lines, osteosarcoma cells were infected with the viral supernatant according to the manufacturer’s protocol. Transfected cells were incubated for 48 h prior to selection of stable transfectants by addition of puromycin (2.5 μg/ml) for two days in selective medium, then those stably transfected cell lines can be obtained (Supplementary Fig. S2).

### Confocal Fluorescence Microscopy

MG63 U2OS and 143B cells were digested, counted, and seeded into coverslips within 12-well plates at 48 h before the analysis. After 48 h of incubation at 37 °C,5% CO_2_, osteosarcoma cells were treated with 5 μmol/L cisplatin for 24 h. Untreated cells were regarded as control group cells. After three washes with PBS, 50 nmol/L mitochondrial probe Mito Tracker-deep Red (Invitrogen) was added, and the cells were incubated for 30 min at 37 °C, 5% CO_2_. Next, the cells were fixed with 4% paraformaldehyde for 30 min and permeabilized with 0.3% Triton X-100 (Beyotime) for 20–30 min. After blocking with goat serum (Beyotime) for 30 min, the slides were incubated with anti-TERT primary antibody (1:100) at 4 °C overnight. Then, the slides were washed three times with PBS, and incubated with Alexa Fluor-488 anti-rabbit IgG (Invitrogen) (1:1000) for 1 h at room temperature. Finally, 4′-6-diamidino-2-phenylindole was added, and the slides were incubated for another 10 min then observed using confocal microscopy. The images were analyzed using ImageJ.

### Apoptotic cell analysis

Percentage of apoptotic cells were assessed by FACScalibur flow cytometry (Becton Dickinson). Sample cells for apoptosis assay were collected by trypsin without EDTA, washed twice with PBS, stained by 5 uL PI and 5 uL FITC-Annexin V in 100 uL binding buffer for 15 minutes in dark (Ebioscience). Fluorescent emissions were collected through FL1 band-pass filter for FITC-Annexin V, FL2 for PI.

### Flow cytometric analysis of intracellular ROS

Cells were loaded with 5-(and-6)-chloromethyl-2-,7-dichlorofluorescindiacetate (DCHF-DA) for cellular ROS measurement as described in Indran’s reports^27^. In brief, sample cells were harvested and washed with 1 x PBS, followed by incubation with 5 mmol/L DCHF-DA for 15 minutes at 37 °C in the dark. Sample cells were then washed in 1 x PBS, resuspended in plain MEM, and analyzed by flow cytometry (Becton Dickinson). 8000 events were analyzed at least.

### Assessment of intracellular GSH:GSSG levels

The glutathione assay was optimized from the protocol by Hissin and Hilf’s reports^41^. Reduced and oxidized glutathione (GSH and GSSG) and N-ethylamide (NEM) solution were obtained from Sigma Aldrich. Cells (1 × 10^6^) were seeded over 2 days in a 6-well plate before treatment. Following with cisplatin treatment, the cells were washed once with chilled PBS. Three hundred sixty microliters of chilled trichloroacetic acid (6.5%) was added to each well and the plates were left at 4 °C for 10 minutes. o-Phthalaldehyde (Sigma Aldrich) solution was freshly prepared in reagent grade absolute methanol. After 30 minutes of incubation in the dark, the plates were excited at 350 nm and fluorescence emission was detected at 420 nm with POLARstar OPTIMA microplate reader (BMG Labtech).

### Glutathione peroxidase and reductase assays

The glutathione peroxidase (GPx) assay was optimized from the protocol provided by the manufacturer (Sigma Aldrich), and cumene hydroperoxide was used as the substrate to measure the total GPx activity. NADPH and cumene hydroperoxide were obtained from Sigma Aldrich. The activity was measured by reading the absorbance at 340 nm once every minute for 15 minutes in a microplate reader (Tecan Spectrofluoroplus). The glutathione reductase (GR) assay was performed as described in the Cayman glutathione reductase assay kit (Cayman Chemical Company).

### Assessment of cytochrome c oxidase activity

Cells were dissolved in 2 ml of COX buffer for 10 min at 4 °C. The cells were then resuspended with 1 ml of COX buffer and incubated for 10 min at 4 °C before being subjected to homogenization for 10 passages and the cell lysate was spun for 10 min at 4 °C. The supernatant was then centrifuged for 10 min at 4 °C. The resultant pellet is the purified mitochondria fraction, containing COX. The pellet was dissolved of pre-warmed potassium phosphate buffer at pH 7.2 and incubated at room temperature (RT) for 3 min. COX activity was measured by monitoring the oxidation of reduced cytochrome c by spectrophotometric analysis at 550 nm for 2 min at 37 °Cusing spectrophotometer.

### Isolation of mitochondrial fractions

Cells were harvested, washed once with ice-cold 1x PBS and pelleted by centrifugation at 1200 rpm for 5 min at 4 °C. Pellet was resuspended in 10 volumes of extraction buffer containing a cocktail of protease inhibitors and incubated on ice for 30 min. After incubation, cells were homogenized with a manual homogenizer for 20 passages and then centrifuged at 1000 g for 10 min at 4 °C. The supernatant obtained was centrifuged at 13,000 g for 20 min at 4 °C to pellet down the mitochondria fraction. The fractions were then subjected to SDS-PAGE and western blotting.

### Assessment of caspase 3 activity

Caspase 3 assay kit was obtained from Sigma Aldrich. All transfected osteosarcoma cells were induced apoptosis in a cell suspension (1 × 10^7^) by addition of cisplatin to a final concentration of 5 μmol/L. The induced cells and the control cells were pelleted by centrifugation at 600 x g for 5 minutes at 4 °C. Supernatant were removed by gentle aspiration and wash the cell pellets once with PBS. The cells were centrifuged and suspended in 1 x lysis buffer at a concentration of 100 µl per 10^7^ cells, incubated on ice for 15 minutes and 4 °C for 15 minutes. After preparation of cell lysates from apoptotic cells, assessment of caspase 3 activity was followed as instruction.

### Animals and transplantation Assay

To determine the *in vivo* tumorigenicity, BALB/C nude mice about 4 weeks old were purchased and maintained at the Wuhan University Center for Animal Experiment. The care and use of animals has been reviewed and approved by the Institutional Animal Care and Use Committee (IACUC) (approval number S01315022l). Osteosarcoma cells (1 × 10^6^) transfected with the TERT-WT, TERT-CI, TERT-siRNA or control construct were counted by trypan blue staining, and suspended in 10 μL of 50% Matrigel/PBS. The mice were randomly divided into four groups (TERT-WT, TERT-CI, TERT-siRNA and Control), with six mice in each group. Tumors were monitored as long as 4 weeks. Data were accumulated from at least three independent experiments.

### qRT-PCR

Total RNA was extracted using the RNeasy Plus Mini Kit and the concentration and purity was determined using an ND-1000 spectrophotometer. Reverse transcription was performed using the TaqMan Reverse Transcription Reagents. Quantitative real-time PCR reactions were set up in triplicate and performed on a 7900 PCR machine using SYBR Green PCR Master Mix. Conditions used for amplification of cDNA fragments were as follows: 95 °C for 5 min, 40 cycles of amplification −95 °C for 20 sec, 60 °C for 1 min. Gene expression levels were calculated using the 2^−ΔΔCt^ method and normalized to the β-actin. Forward and reverse primers for TERT and β-actin were 5′-GGATTGCCACTGGCTCCG, 5′-TGCCTGACCTCCTCTTGTGAC; and 5′-GTCCACCGCAAATGCTTCTA, 5′-TGCTGTCACCTTCACCG TTC, respectively.

### Western-Blot analysis

Proteins were extracted with Protein Lysis Buffer. Lysates were centrifuged at 10000 g at 4 °C for 10 min, and supernatants collected. Before transferred to nitrocellulose membranes (Amersham, Chalfont), the proteins (50 μg) were separated by SDS-PAGE. The membranes were blocked in 5% non-fat dry milk and were subsequently incubated overnight at 4 °C with antibodies, purchased from Abcam: TERT (ab94523, 1 μg/ml), Bax (ab53154, 1/1000), Bcl-2 (ab32124, 1/1000), Bcl-xl (ab32370, 1/1000), and β-actin (ab8227, 1/1000) (as an internal standard). The membranes were incubated with secondary antibodies-goat anti-mouse IgG-horseradish peroxidase (HRP) (ab191866, 1/1000) for 1 h at room temperature after washed 3 times with Tris-buffered saline containing 0.1% Tween-20 (TBST). The density of the bands was quantified using Quantity One software (Bio-Rad Laboratories, Inc.) in three experiments.

### Statistical analysis

All experiments were performed at least 3 times for statistical significance. Numerical data were expressed as mean ± SD. Statistical analysis was performed using the paired Student’s t test considering the variances unequal. P < 0.05 was considered significant.

## Electronic supplementary material

Supplementary Information
