# Supplementary material for: Telomerase reverse transcriptase promotes chemoresistance by suppressing cisplatin-dependent apoptosis in osteosarcoma cells
Source: Sci Rep. 2017 Aug 1;7:7070. doi: 10.1038/s41598-017-07204-w (PMC5539325; doi:10.1038/s41598-017-07204-w)
Supplement: Supplementary file 1 — Supplementary Information [file 41598_2017_7204_MOESM1_ESM.pdf]

Supplementary Information

Supplementary Figures S1-S2

Telomerase reverse transcriptase promotes chemoresistance by suppressing cisplatin-dependent apoptosis in osteosarcoma cells

Zhengpei Zhang1, Ling Yu1#, Guo Dai1, Kezhou Xia1, Gaiwei Liu1, Qi Song1, Chunjie Tao1, Tian Gao2, Weichun Guo1#

Supplementary Fig.S1

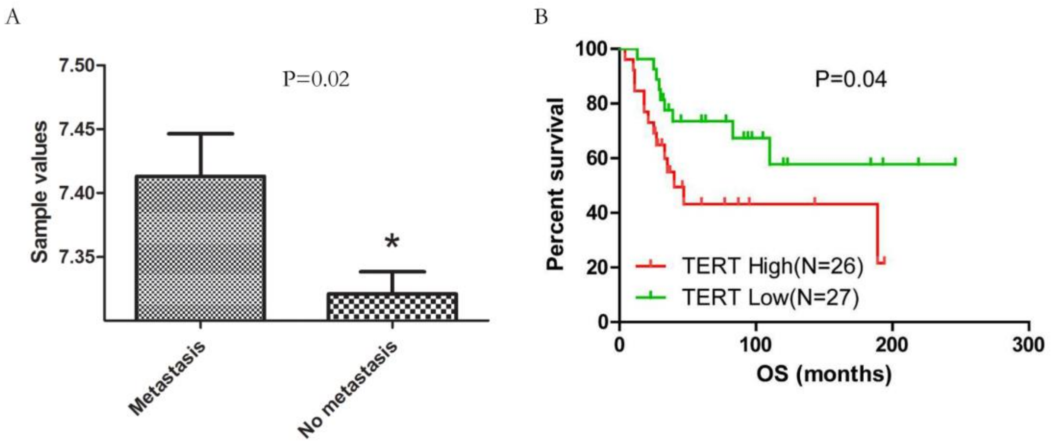

Supplementary Fig.S1. Publicly datasets and Analysis. Publicly available datasets were analyzed to assess the potential role of TERT in osteosarcoma. GSE21257 contains expression data from pre-chemotherapy biopsies of osteosarcoma patients who developed metastases within 5yrs (n=34) and those who did not develop (n=19). GEO2R was used to compare two or more groups of samples in order to identify genes that are differentially expressed across experimental conditions. (A) Expression value of TERT was significantly higher in metastasis patients than in no-metastasis patients. (B) Survival analysis of the same datasets showed that TERT High patients presented with poor prognosis.

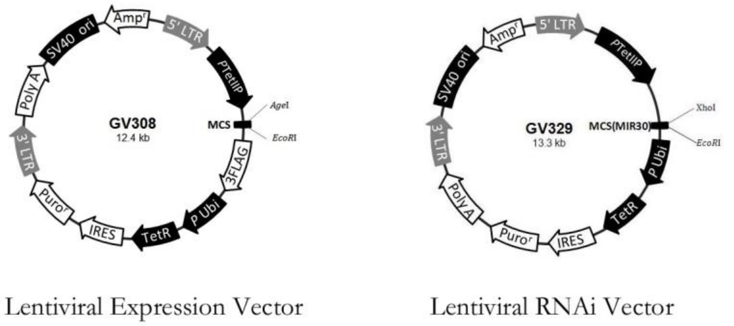

Supplementary Fig.S2. An illustration of Lentiviral Expression Vector and Lentiviral RNAi Vector.
